# Dual mobility acetabular components for revision THA

**DOI:** 10.1007/s10195-015-0347-x

**Published:** 2015-02-21

**Authors:** Michael B. Cross

**Affiliations:** Department of Orthopedic Surgery, Hospital for Special Surgery, 535 East 70th Street, New York, NY 10021 USA

Recurrent instability after primary and revision total hip arthroplasty (THA) is a disastrous complication for the surgeon and the patient. Dislocation after revision total hip arthroplasty has been reported to be as high as 20 % in some series [1]. Patients who suffer from recurrent dislocations are challenging because historical treatment options, including constrained liners, have had disappointing results [2]. Dual mobility acetabular cups were initially introduced to reduce dislocation rates after primary total hip arthoplasty [3]. While dual mobility acetabular components have been shown to improve stability in primary THA, few studies have examined the outcomes of dual mobility bearings in revision THA for persistent dislocation [4].

The current study by van Heumen et al. [5] was a retrospective cohort study with 49 consecutive patients (50 hips) that underwent an isolated acetabular revision with a dual mobility cup (Avantage; Biomet, Warsaw, IN, USA) for recurrent instability with an average follow-up of 29 months (12–66 months) [3]. The cohort of patients was challenging, as 30 patients (60 %) had more than two surgeries. However, despite a challenging cohort of patients, no post-operative dislocations occurred during follow-up period; however, three hips were revised, most commonly for infection. Overall, the survival rate for dislocation after 56 months was 100 % and 93 % for all cause revision. Although this study does not have long-term follow-up results or any functional outcome data, it does demonstrate excellent 5 year survival rate with a dual mobility cup in revision THA for recurrent instability.

While I commonly use dual mobility bearings for patients with a high risk of dislocation and for revisions for recurrent instability, my personal results have been less impressive than van Heuman et al. and Mohammed et al. In this challenging group of patients, I have seen patients who still suffer from recurrent dislocations, despite using a dual mobility bearing. I also have seen intraprosthetic dissociation, particularly using smaller head sizes (e.g., 22 mm) and when the implant company of the femur differs from the implant company of the dual mobility acetabular component. Still, the current study highlights that a dual mobility bearing may be a great option for patients who require revision for recurrent instability. However, it goes without saying that this remains a very challenging group of patients, and using a dual mobility bearing seems to improve the risk of dislocation compared to historical treatment options but the risk is not entirely eliminated.
